# Multi-Omics Decoding of Potential Microbial–Genetic Synergy Underlying Polysaccharide and Glycosidic Polymer Biosynthesis in Two Cultivars of *Lilium brownii* var. *viridulum* Baker

**DOI:** 10.3390/metabo15110712

**Published:** 2025-10-30

**Authors:** Tao Chang, Yajie Xue, Fan Liu, Ran Zheng, Zaiqi Zhang, Qinfang Zheng, Putao Wang

**Affiliations:** 1 Key Laboratory of Dong Medical Research of Hunan Province, Biomedical Research Institute, Hunan University of Medicine, Huaihua 418000, China; changtao@hnmu.edu.cn (T.C.);; 2 School of Pharmaceutical Sciences, Hunan University of Medicine, Huaihua 418000, China

**Keywords:** polysaccharide and glycosidic polymer, multi-omics, microbial–genetic synergy, *Lilium brownii* var. *viridulum* Baker ‘Xuefeng’

## Abstract

**Background:** The accumulation of glycosidic polymers in *Lilium brownii* var. *viridulum* Baker (*Lv*) bulbs fundamentally governs the nutritional and medicinal properties. **Methods:** In this study, metabolomic, transcriptomic, and microbiome analyses were integrated to elucidate the differential mechanisms of glycoside accumulation between the elite ‘Xuefeng’ (*Lv*, X) and ‘Longya’ (*Lv*, L), each comprising three biological replicates. **Results:** The results demonstrate significantly elevated diversity and abundance of glycosides in X bulbs, with glucose derivatives constituting the predominant fraction. Differential expression genes (DEGs) associated with carbohydrate metabolism were primarily enriched in starch/sucrose metabolism and amino sugar metabolic pathways. Planctomycetes in rhizospheric soil, combined with Acidobacteriia and Rhodanobacteraceae in non-rhizospheric soil, were identified as key microbial taxa associated with glycoside accumulation. Variation partitioning analysis (VPA) revealed that synergistic genetic microbiota–host interactions collectively accounted for 86.8% of the metabolic variance. **Conclusions:** Consequently, X exhibits superior potential as a medicinal/edible cultivar and as a breeding material due to its enhanced biosynthesis of glycosidic polymers. This work, for the first time, systematically deciphers the regulatory framework of glycoside accumulation in *Lv* bulbs, highlighting microbiota–host synergy, and provides critical insights for the refining of biosynthetic pathways and targeted crop enhancement.

## 1. Introduction

*Lilium brownii* var. *viridulum* Baker, whose bulbs are rich in various bioactive glycosides, exhibits significant pharmacological effects, including antioxidant, immunomodulatory, and anticancer activities, making it a vital dual-purpose resource for both medicinal and food applications in China [1,2]. The content and composition of glycosidic substances in *Lv* bulbs are critical determinants of the final product’s quality. Specifically, polysaccharides and glycosidic derivatives directly influence its sensory properties, processing quality, as well as nutritional and health benefits [3,4,5]. Given the diversity among *Lv* varieties, the saccharide content in their bulbs exhibits significant variation between cultivars [6,7]. The ‘Xuefeng’ germplasm, a specific *Lv* originating from the Xuefeng Mountain region at 27°42′ N, 110°35′ E, between the cities of Huaihua and Shaoyang, Hunan Province, China, is notably rich in secondary metabolites, representing a potential elite genetic resource for breeding new varieties. Research indicates that the accumulation of secondary metabolites in *Lv* bulbs is governed by intricate biosynthetic and regulatory networks and is closely coupled to the buildup of glycosidic polymers [8,9]. Consequently, a deep understanding of the regulatory mechanisms controlling sugar metabolism in different *Lv* bulbs is crucial for enhancing the quality of this dual-purpose medicinal/food crop.

Plant rhizosphere microbial communities influence the accumulation of secondary metabolites produced by the host through pathways including nutrient cycling [10,11,12], hormone signaling transduction [13,14,15], and host immune modulation [16,17]. Recent research has revealed that microbes dynamically shape the microecology of the rhizosphere by synthesizing extracellular polysaccharides or secreting glycoside hydrolases, thereby establishing cross-kingdom sugar signaling networks—that is, communication between different kingdoms of life, such as bacteria and plants—that regulate the host’s sugar metabolism pathways [18,19].

This regulation primarily manifests through the modification of polysaccharide side-chain configurations by enzyme systems such as *β*-glucosidase [20,21] or the activation of key glycosyltransferase gene expression, exemplified in plants by sucrose synthase (SuSy) genes [22,23]. While mechanistic insights for staple food crops like rice [24,25,26] and maize [27,28,29] have advanced, it remains unclear which specific microbes influence the synthesis and accumulation of glycosidic substances in the economically significant *Lilium brownii* var. *viridulum* Baker, whose bulbs are primarily valued for their glycoside content. Furthermore, systematic studies comparing glycoside profiles across different *Lv* bulb materials are currently lacking. This knowledge gap constitutes a critical bottleneck for quality improvement through microecological regulation, severely limiting targeted rhizosphere microbiome manipulation to optimize glycosidic polymer quality in *Lv* bulbs.

This study focuses on the regulatory effects of rhizosphere microbial community differences between the ‘Xuefeng’ (X) and ‘Longya’ (L, a common cultivar used as a control in Lilium research) of *Lv* on glycoside metabolism in their bulbs. We employed an integrated multi-omics strategy, combining metabolomics, transcriptomics, and microbiome analysis, to systematically compare differences in microbial community structure (rhizosphere and non-rhizosphere soils), bulb glycoside metabolite profiles, and key gene expression patterns between the two lines. This comprehensive approach examines the soil microbiome, genetic background, and metabolic profiles to elucidate potential links between glycoside accumulation, genetics, and the soil microbial community. Specifically, the study focuses on (1) characterizing the differences between L and X regarding bulb glycosides, related genes, and rhizosphere/non-rhizosphere soil microbes; (2) identifying key factors contributing to divergent glycoside accumulation in the two *Lv* bulbs by integrating microbial, genetic, and metabolite data; and (3) constructing a holistic regulatory network integrating microbiome, genetics, and metabolism to clarify the mechanisms underlying glycoside accumulation in X and L. Significantly, this work represents the first systematic investigation into glycoside variation and accumulation in *Lv* bulbs, providing a foundational reference for developing targeted microbiome-based strategies to regulate glycosidic polymer biosynthesis in this medicinal/food crop.

## 2. Materials and Methods

### 2.1. Sample Collection

Bulbs of ‘Xuefeng’ (X) and ‘Longya’ (L) were collected from current-year plants at the *Lv* germplasm nursery in Beidouxi Town, Xupu County, Hunan Province, China (110°33′56.6136″ E, 27°37′39.5472″ N). The local soil is a slightly acidic loam. After thorough cleaning, six replicate samples of 4 g of bulb tissue each were pooled. The pooled sample was then immediately flash-frozen in liquid nitrogen, typically within five seconds, for metabolomic and transcriptomic analyses. Rhizosphere soil (R) was collected from root surfaces using sterile brushes. Non-rhizosphere soil (M) was sampled via a five-point method: surface debris was removed from a 1–6 cm annular zone surrounding the roots, followed by excavation of subsurface soil (to a depth of 2–5 cm). Both soil types were cleared of visible impurities, sieved through a 20-mesh screen, transferred to centrifuge tubes, and immediately flash-frozen in liquid nitrogen for storage.

### 2.2. Metabolomic Analysis

#### 2.2.1. Metabolite Extraction and Chromatographic Analysis

Metabolite profiling of cultivar bulbs was performed by Wuhan MetWare Biotechnology Co. Ltd. (Wuhan, China) using an LC-ESI-MS/MS system (HPLC, Shim-pack UFLC SHIMADZU CBM30A) equipped with a Waters ACQUITY UPLC HSS T3 C18 column (1.8 μm, 2.1 mm × 100 mm). The mobile phase consisted of solvent A (water containing 0.04% acetic acid) and solvent B (acetonitrile containing 0.04% acetic acid). The gradient elution program was as follows: 0 min, 100% A; 11.0 min, 5% A; 12.0 min, 5% A; 12.1 min, 95% A; and 15.0 min, 95% A. The flow rate was 0.40 mL/min, the column temperature was maintained at 40 °C, and the injection volume was 2 μL.

#### 2.2.2. Metabolite Identification, Quantification, and Data Processing

Metabolites were identified using the in-house MetWare database. Quantification was performed using the multiple reaction monitoring mode on a triple quadrupole mass spectrometer. The signal intensity of characteristic ion pairs for each metabolite was detected. Chromatographic peak integration and correction were conducted using MultiQuant software (3.0.2, SCIEX, Framingham, MA, USA). The peak area of each chromatographic peak represented the relative content of the corresponding metabolite. Metabolite data were processed and analyzed using MetaboAnalyst v4.0. Post processing, multivariate data analysis was applied to examine the three-dimensional matrix comprising sample descriptions, *m*/*z* pairs, and retention times. To compare metabolite-level differences across samples, metabolite peaks in the mass spectra were calibrated based on retention time and peak shape.

#### 2.2.3. Differential Metabolite Screening and Functional Annotation

Differential metabolites were visualized using volcano plots [30]. Principal component analysis (PCA) was employed for sample clustering [31]. Significantly differential metabolites were screened based on the following criteria: fold change (FC) < 0.5 or FC > 2 and variable importance in projection (VIP) > 1. Differential metabolites were mapped to relevant metabolic pathways using the KEGG database. Pathway enrichment *p*-values were calculated, with *p* < 0.05 indicating a significantly perturbed pathway.

### 2.3. Transcriptomic Analysis

#### 2.3.1. RNA Extraction

Total RNA was extracted from the bulb ecotypes using the TaKaRa MiniBEST Plant RNA Extraction Kit (TaKaRa, Beijing, China). RNA purity, concentration, and integrity were assessed using 2% agarose gel electrophoresis, a NanoPhotometer spectrophotometer, a Qubit 2.0 Fluorometer, and an Agilent 2100 Bioanalyzer with the RNA Nanochip. Libraries were constructed using RNA samples that passed quality control. mRNA was purified from total RNA using oligo (dT)-attached magnetic beads. The purified mRNA was then fragmented using fragmentation buffer.

#### 2.3.2. Library Construction and Sequencing of Transcriptomic

First-strand cDNA was synthesized using the fragmented RNA as a template and random hexamer primers. Second-strand cDNA synthesis was subsequently performed using buffer, dNTPs (dUTP, dATP, dGTP, and dCTP), and DNA polymerase I. The double-stranded cDNA (ds cDNA) was purified using AMPure XP beads. The purified ds cDNA underwent end repair, A-tailing, and adapter ligation. Following size selection with AMPure XP beads, the resulting fragments were enriched by PCR to generate the final cDNA libraries. Library concentration and insert size were measured using the Qubit 2.0 Fluorometer and Agilent 2100 Bioanalyzer, respectively. The effective library concentration was accurately quantified via q-PCR to ensure library quality. After passing quality control, libraries were pooled according to the target sequencing data volume and sequenced on the Illumina HiSeq platform.

#### 2.3.3. Differential Gene Screening and Functional Annotation

DEGs between the two ecotypes were identified using the DESeq2 statistical method. The resulting *p*-values were adjusted using the methods of Hu [32] and Ammah [33]. DEGs were screened based on the criteria: |Log_2_(FC)| > 1 and adjusted *p*-value (padj) < 0.05. DEGs were annotated using the Kyoto Encyclopedia of Genes and Genomes (KEGG) database to map them onto metabolic and signal transduction pathways, thereby elucidating their roles in cellular metabolism and signaling [34]. Gene Ontology (GO) annotation was employed to categorize DEGs into three functional domains—biological process, cellular component, and molecular function—providing insights into gene localization, biological involvement, and molecular functions [35].

### 2.4. Soil Metagenomic Analysis

#### 2.4.1. DNA Extraction

Metagenomic analysis of microbial communities in rhizosphere soil (R) and non-rhizosphere soil (M) from *Lv* bulbs was conducted by Biomarker Technologies Corporation (Beijing, China). Total genomic DNA was extracted using a commercial soil genomic DNA extraction kit. DNA integrity was assessed via 1% agarose gel electrophoresis, while concentration and purity were determined using a NanoDrop 2000 spectrophotometer (Thermo Scientific, Waltham, MA, USA). Specific PCR amplification targeting different microbial taxa was performed. Amplicons from triplicate reactions per sample were pooled, and target bands were excised from 2% agarose gels and purified using the AxyPrep DNA Gel Extraction Kit. Purified product concentration was quantified using a Quantus™ Fluorometer.

#### 2.4.2. Library Construction and Sequencing of Soil Metagenomic

Sequencing libraries were prepared using the NEXTflex™ Rapid DNA-Seq Kit. The workflow included end repair, A-tailing, adapter ligation, size selection with magnetic beads to remove adapter dimers, PCR amplification for library enrichment, and final PCR product purification using magnetic beads. Final libraries were subjected to paired-end sequencing on either the Illumina MiSeq PE 300 platform or the Illumina NovaSeq PE 250 Statistical analyses were performed in R (v4.1.3). The results were visualized primarily using the ggplot2 or MetWare Cloud platform packages. Alpha diversity was assessed according to Finn [36]. Beta diversity analysis was performed according to Glassmire [37]. Permutational multivariate analysis of variance (PERMANOVA) was conducted as described by Shen [38]. Linear discriminant analysis effect size (LEfSe) was implemented based on Chang [39]. Microbial functional potential in soil samples was predicted using the method outlined by Singavarapu [40]. PCA and Hierarchical Cluster Analysis (HCA) were employed for sample clustering [41]. VPA was executed using the vegan package and Mantel tests were performed using the linkET package [42]. Microbial genetic metabolic networks were visualized using Cytoscape (v3.5) [43].

## 3. Results and Analysis

### 3.1. Metabolic Differences Between Glycosides in Two Cultivars

**Figure 1:**
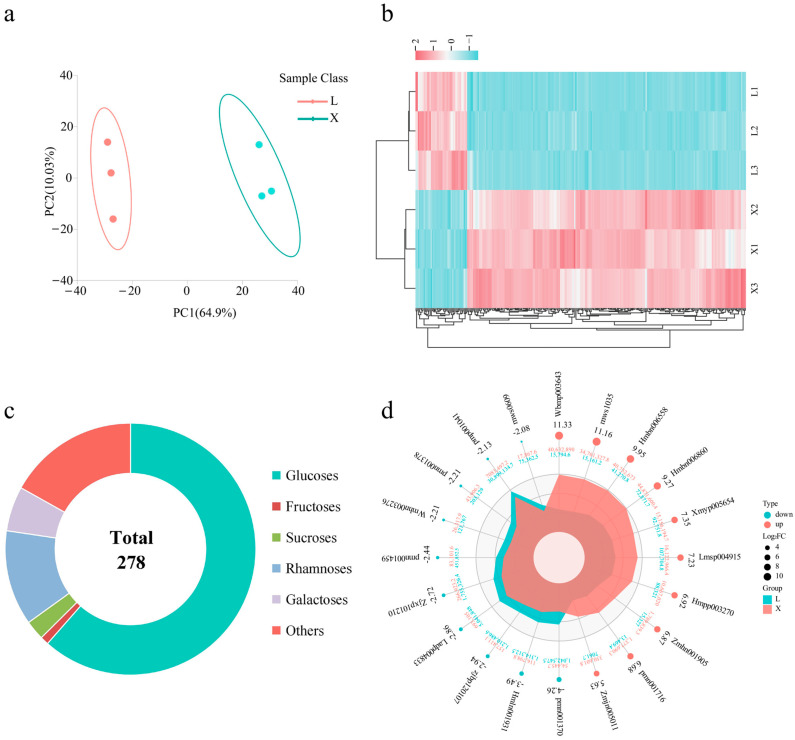
Differences in glycoside accumulation between the two bulbs. (**a**) PCA of bulb metabolomics data, (**b**) heatmap of glycoside-type DAMs, (**c**) classification of glycoside-type DAMs by glycosyl composition, and (**d**) radar plot of the top 10 and bottom 10 ranked glycoside-type DAMs. L: ‘Longya’, X: ‘Xuefeng’.

PCA of the metabolomic data from the two bulbs (Figure 1a) revealed distinct clustering into two groups, indicating reliable metabolomic results with minimal intra-group variation and significant metabolic differences between the varieties. Figure S1 displays the differentially accumulated metabolites (DAMs) identified between the X and L, totaling 977 DAMs, with 352 upregulated and 125 downregulated. Based on the metabolomic results, DAMs were ranked by their log_2_FC values, revealing the differences in monosaccharide content as shown in Figure S2. Targeted screening for glycoside-type DAMs yielded 278 metabolites (Table S1), with 234 upregulated and 44 downregulated (Figure 1b). KEGG enrichment analysis of these 278 DAMs identified 20 significantly enriched metabolic pathways (Figure S3). The top five pathways were glucosinolate biosynthesis, cyanoamino acid metabolism, anthocyanin biosynthesis, valine, leucine and isoleucine degradation, and indole alkaloid biosynthesis. Classification of sugar derivatives by their glycosyl moieties (Figure 1c) ranked glucose, rhamnose, fructose, and other sugars as the most abundant. Classification by the secondary metabolite aglycones (Figure S4) yielded flavonoids, phenols, steroids, saccharides, lignans, glycerides, and monoterpenoids, which were identified as the predominant types. The top 10 upregulated and downregulated glycoside-type DAMs identified by metabolomics are presented in Figure 1d. The fold change (FC) range for the top 10 upregulated DAMs was 5.63 to 11.33, while it was −4.26 to −2.08 for the top 10 downregulated DAMs. These results demonstrate significant differences in glycoside accumulation between the two bulbs. The X cultivar exhibited a greater diversity and abundance of glycosides compared to L, predominantly comprising glucose and its derivatives.

### 3.2. Transcriptome Profiling Reveals Gene Expression Differences Between Bulbs of Two Cultivars

**Figure 2:**
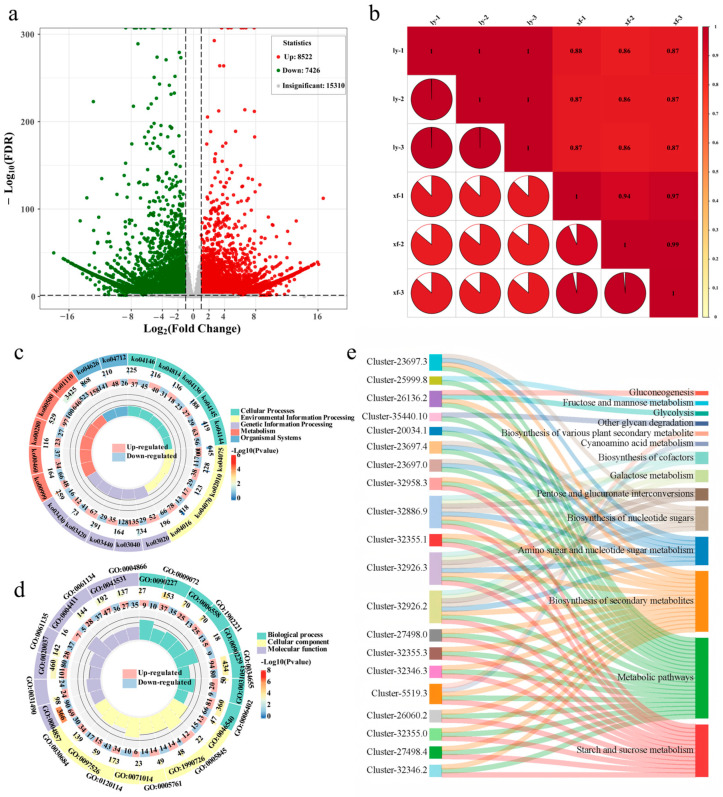
Differential transcriptome analysis of two ecotypes’ bulbs. (**a**) Volcano plot of DEGs, (**b**) intra-group clustering correlation analysis of biological replicates, (**c**) KEGG enrichment analysis, (**d**) GO enrichment analysis, and (**e**) Sankey diagram of functional enrichment for key genes. ly (L): ‘Longya’, xf (X): ‘Xuefeng’.

To identify key DEGs associated with glycoside accumulation in the bulbs of the two ecotypes, transcriptome analysis was performed on mature bulbs. Quality control metrics confirmed data integrity (Table S2). This analysis identified 15,948 DEGs, comprising 7426 upregulated and 8522 downregulated genes (Figure 2a). Applying stringent criteria (|log_2_FPKM| ≥ 2, adjusted *p*-value < 0.05), we identified 642 DEGs directly involved in carbohydrate metabolism in both X and L (Table S3). PCA showed clear separation between the two varieties (Figure S5). Biological replicates exhibited strong intra-group correlations (R > 0.85, Figure 2b), confirming high reproducibility within groups and significant transcriptional differences between groups. KEGG and GO enrichment analyses were performed on genes potentially encoding glycoside accumulation pathways. KEGG analysis assigned the enriched DEGs to nine subcategories (Figure 2c). Key glycoside-related pathways included starch and sucrose metabolism. The top enriched pathways (Figure S6) were amino acid biosynthesis (4.91%), starch and sucrose metabolism (4.17%), and phenylpropanoid biosynthesis (2.41%). GO analysis categorized enriched terms as biological process, cellular component, and molecular function (Figure 2d). Significantly enriched terms (Figure 2d and Figure S7) included the nucleobase-containing compound catabolic process (2.2%), heme binding (2.18%), the RNA catabolic process (1.97% and 1.86%), and enzyme inhibitor activity (1.91%). To pinpoint key genes regulating glycoside synthesis, the top 10 upregulated and top 10 downregulated DEGs linked to glycoside metabolism were selected (Table S4). Functional enrichment of these 20 genes revealed, nine in starch and sucrose metabolism, four in amino sugar and nucleotide sugar metabolism, three in pentose and glucuronate interconversions, and the remaining four in fructose and mannose metabolism, glycolysis/gluconeogenesis, cyanoamino acid metabolism, and other glycan degradation pathways, respectively (Figure 2e). Based on the fold change magnitude, the top four upregulated and top four downregulated genes from this set were identified as candidate genes for subsequent analysis.

### 3.3. Diversity and Composition of Soil Microbiota Associated with Two Cultivars

**Figure 3:**
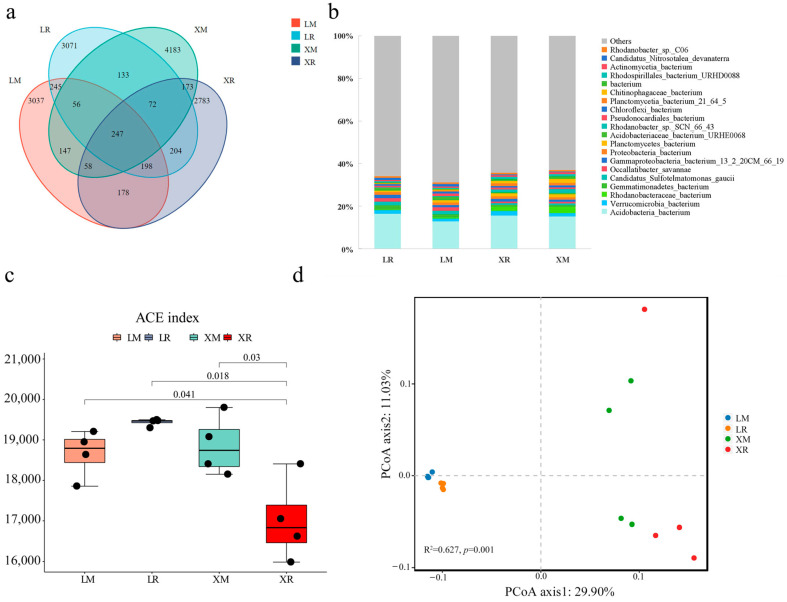
Differences in soil microbial communities between two *Lv*s. (**a**) Venn diagram of microbial taxa across soil samples, and (**b**) bacterial composition at the species level. (**c**) Alpha diversity analysis between two *Lv*s. (**d**) PCoA of beta diversity between two *Lv*s. L: ’Longya’, X: ’Xuefeng’. R: Rhizosphere soil, M: Non-rhizosphere soil.

To identify microbial differences in the rhizosphere and non-rhizosphere soils of the two *Lv*s, metagenomic analysis was performed on four soil types. This revealed 247 core microbial taxa shared across all soils, though with significant variations in relative abundance (Figure 3a). Taxonomic annotation showed similar dominant bacterial community structures across soil treatments. Bar plots depict the relative abundances of the top 20 species, as well as phyla and genera (Figures S8 and S9). At the species level (Figure 3b), the predominant known bacterial taxa were Acidobacteria (9.19%), Rhodanobacteraceae (1.05%), and Verrucomicrobia (0.90%). Alpha diversity analysis (Figure 3c) revealed significantly higher indices in L rhizosphere soil than in X rhizosphere soil, while the opposite pattern was observed in the non-rhizosphere soil (*p* < 0.01). The rhizosphere soil of L exhibited higher alpha diversity than that of X, while the opposite trend was observed in non-rhizosphere soil. Principal Coordinates Analysis (PCoA) of beta diversity showed distinct clustering among the four sample groups (Figure 3d), indicating significant differences in microbial enrichment between rhizosphere and non-rhizosphere soils for both varieties, demonstrating a pronounced rhizosphere effect. The paired PERMANOVA (Figure 3d) results also showed that the *p*-values between the two groups of materials in both soils were 0.001, which was less than 0.002, indicating that the microbial communities in various soils exhibit regional specificity and good intra-sample reproducibility.

**Figure 4:**
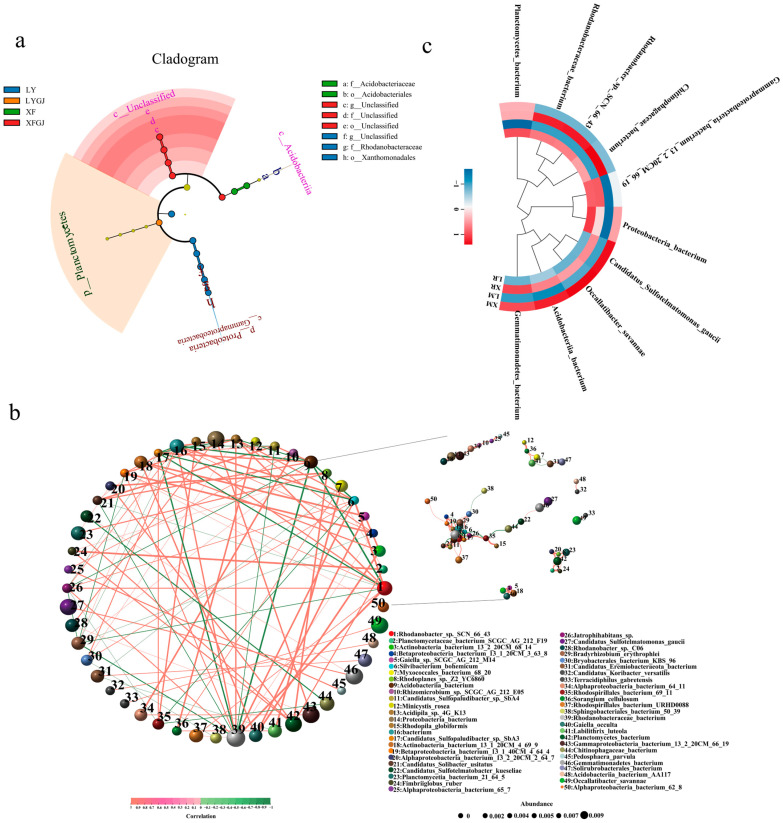
Screening of potentially glycoside metabolism-associated key microbes in rhizosphere and non-rhizosphere soils of two *Lv*s, (**a**) LEfSe analysis of microbial composition across four soil types, (**b**) co-occurrence network analysis of the top 50 most abundant microbial taxa, and (**c**) relative abundance analysis of key candidate microbes. L: ‘Longya’, X: ‘Xuefeng’. R: Rhizosphere soil, M: Non-rhizosphere soil.

To distinguish rhizosphere and non-rhizosphere soil microbes exhibiting material-driven variations between the two *Lv*s, we performed LEfSe analysis coupled with LDP evolutionary cladograms to identify statistically significant microbial taxa across four soil types. The results showed that ‘Xuefeng’ Rhizosphere (XR) soils were enriched in Acidobacteriia (class), alongside one unknown class, one unknown order, one unknown family, and one unknown genus. LR soils showed Planctomycetes (phylum) dominance. ‘Xuefeng’ non-rhizosphere (XM) soils exhibited an increased abundance of Acidobacteriales (order) and Acidobacteriaceae (family). LM soils were characterized by enrichment of *Bacteria* (kingdom), Proteobacteria (phylum), Gammaproteobacteria (class), Xanthomonadales (order), Rhodanobacteraceae (family), and one unknown genus (Figure 4a).

To further investigate whether the biological functions of differentially enriched bacteria are associated with glycoside biosynthesis, we performed functional enrichment analysis on the identified microbial taxa using the KEGG database (Figure S10). Among the top 10 enriched pathways across the four soil types, carbohydrate metabolism and glycan biosynthesis and metabolism were directly linked to glycoside synthesis. Notably, the average proportion of carbohydrate metabolism enrichment was significantly higher in XR and XM compared to LR and LM. Conversely, glycan biosynthesis and metabolism showed significantly lower enrichment in XR and XM than in LR and LM. Subsequently, we conducted co-occurrence network analysis on the top 50 most abundant microbial taxa (Figure 4b). Based on relative abundance and inter-taxa correlations, the 10 highest-abundance microbes were selected for further comparison between rhizosphere and non-rhizosphere soils of the two *Lv*s. The results demonstrated significant differences (*p* < 0.05) in relative abundance for nine out of the ten selected microbial taxa in rhizosphere soils, with the exception of bacterium_13_2_20CM_66_19 from Gammaproteobacteria. Similarly, in non-rhizosphere soils, nine of ten microbes showed significant abundance variations, excluding bacterium from Planctomycetes (Figure 4c). Based on LEfSe-identified dominant microbial groups, we ultimately selected two taxa (bacterium from Acidobacteriia and bacterium from Planctomycetes) from the rhizosphere soils and four taxa (bacterium from Acidobacteriia, bacterium from Rhodanobacteraceae, bacterium_13_2_20CM_66_19 from Gammaproteobacteria, and bacterium from Proteobacteria) from the non-rhizosphere soils for subsequent functional analysis.

### 3.4. The Relationship Between Soil Microorganisms, Genetic Factors, and Metabolic Regulation in the Two Cultivars

**Figure 5:**
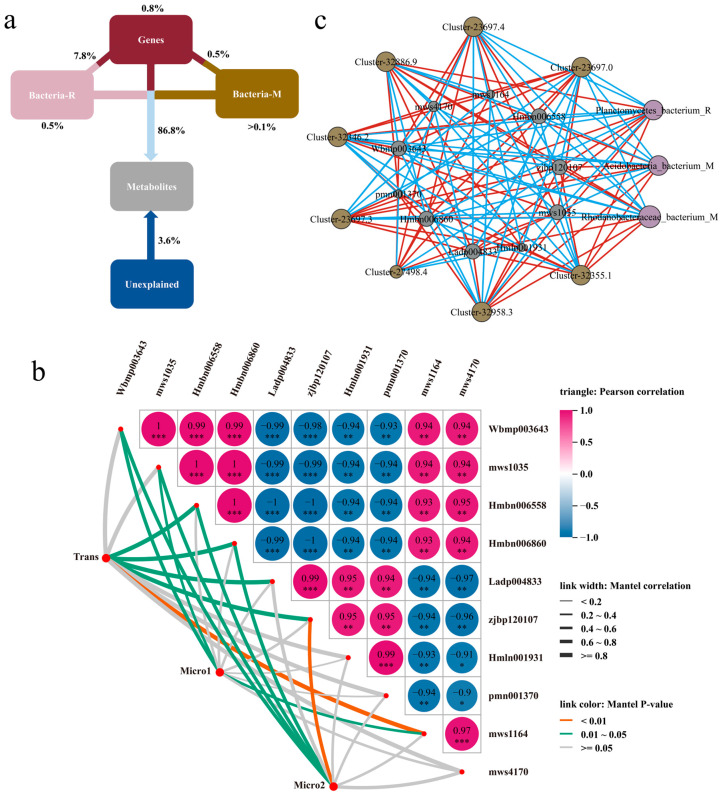
Difference in accumulation of glycosides in the two *Lv*s. (**a**) Combined effects of microbial and genetic factors on metabolites, (**b**) Mantel test for the associations of bacteria and genes with metabolites, and (**c**) microbial–genetic–metabolic regulatory network. Note: * meaning weak correlation, ** meaning moderate Correlation, *** meaning strongly correlated.

To further clarify the influence of microbial and genetic factors, as well as their interactions, on the regulation of the two *Lv*s glycoside metabolites, we conducted VPA on the glycoside metabolites. The results indicated that microbial and genetic factors and their interactions were significantly associated with the metabolite levels of bulb glycosides. The combined effects of genetic factors, rhizosphere soil, and non-rhizosphere soil collectively explained 86.8% of the metabolite variation. Interactions between genetic factors and rhizosphere soil accounted for 7.8% of the variation, while interactions between genetic factors and non-rhizosphere soil explained 0.5%. Genetic factors and rhizosphere soil individually contributed 0.8% and 0.5%, respectively, while non-rhizosphere soil explained less than 0.1%. Additionally, 3.6% of the variation remained unexplained by this three-factor system (Figure 5a). Subsequently, in Mantel tests on the 10 previously identified differential metabolites, eight DEGs and six soil microorganisms revealed significant correlations between genetic factors and five metabolites, rhizosphere soil and seven metabolites, and non-rhizosphere soil and five metabolites (Figure 5b). Furthermore, multi-omics association analysis indicated that bacterium from Planctomycetes in the rhizosphere soil, and bacterium from Acidobacteriia and bacterium from Rhodanobacteraceae in the non-rhizosphere soil, exhibited correlations exceeding 0.75 with both DEGs and DEMs (Figure 5c). Consequently, bacterium from Planctomycetes in the rhizosphere soil, along with bacterium from Acidobacteriia and bacterium from Rhodanobacteraceae in the non-rhizosphere soil, were identified as key microorganisms strongly correlated with glycoside accumulation in *Lv*s.

## 4. Discussion

### 4.1. Breeding Based on Differences in Lv Bulbs’ Glycoside Contents

This study, for the first time, systematically deciphered the regulatory mechanisms governing glycoside accumulation in the bulbs of two *Lv*s using a multi-omics approach. Metabolomic analysis revealed that both the variety and quantity of glycosides, especially polysaccharides and glycosidic polymers, were significantly higher in the bulbs of X compared to those of L (Figure 1), underscoring their potential as bioactive carbohydrate polymers with enhanced functional properties. We identified a total of 977 differential metabolites between the two ecotypes’ bulbs, with glycosides comprising 278 (28%) of these (Figure 1b). Of these 278 glycosides, 234 were upregulated and 44 were downregulated. The significantly upregulated glycosides were predominantly secondary metabolites with health-promoting functions, particularly flavonoids and phenolics, the accumulation of which is closely linked to the medicinal value and antioxidant activity of *Lv*s [44,45], directly underpinning their core quality traits as a dual-purpose crop that can be used in the production of both medicine and food, encompassing edibility, processing suitability, and nutritional or health value. Based on the glycone moiety, most were glucosides or their derivatives. Furthermore, key metabolic pathway analysis indicated specific activation of glucosinolate biosynthesis and anthocyanin synthesis pathways in X bulbs. Previous studies have shown that flavonoid glycoside and glucosinolate accumulation are significantly associated with plant stress resistance and medicinal properties [46,47,48], while activation of the anthocyanin synthesis pathway is often linked to cultivar-specific metabolic regulatory networks [49,50]. These findings provide a pivotal foundation for evaluating *Lilium brownii* var. *viridulum* Baker germplasm resources and for breeding novel cultivars with high added value, as well as offering new insights for elucidating the molecular mechanisms of glycoside biosynthesis in *Lv* species.

### 4.2. Regulatory Role of Genetic Background Differences on Glycoside Synthesis

The two *Lv*s used in this study exhibit substantial genetic background differences. Transcriptome analysis identified 15,948 DEGs between them, with 642 directly involved in carbohydrate metabolic pathways (Figure 2). Enrichment analysis of these genes revealed significant expression differences in pathways directly related to glycoside synthesis, particularly starch and sucrose metabolism, amino sugar and nucleotide sugar metabolism, and pentose and glucuronate interconversions. Genes in pathways such as starch and sucrose metabolism (ko00500) were significantly upregulated in X. These pathways are central to generating glycosyl donors (UDP-glucose) and glycosylation reactions. Their differential gene expression directly regulates the supply of glycoside precursors and the activity of glycosyltransferases in the bulbs. By accelerating UDP-glucose production, these pathways provide precursors for glycosylation, thereby influencing the ultimate accumulation of glycosides [51]. Additionally, GO analysis indicated differences in pathways such as nucleobase-containing compound catabolism and RNA catabolism between the two bulbs (Figure 2d). Monosaccharide content analysis also showed significantly lower ribose levels in X bulbs compared to those of L. We propose this may stem from enhanced ribose metabolism in X. This is potentially due to upregulated activity or expression of key enzymes like ribokinase, leading to accelerated ribose consumption. As ribose is a crucial precursor for nucleotide and certain coenzyme synthesis [52], its reduced content and accelerated utilization indirectly impact cellular energy status and nucleotide pool balance. These changes further feedback to regulate the flux of sugar nucleotide biosynthesis. Specifically, the altered cellular energy status and nucleotide pools resulting from differential ribose metabolism in X induce differential expression of genes encoding key enzymes for sugar nucleotide synthesis. This promotes increased production of glycosyl donors. These abundant glycosyl donors provide an ample substrate for glycosyltransferases, driving the glycosylation of specific secondary metabolites. Integrated analysis of key genes and metabolites revealed that their down-regulated expression significantly enhanced the synthesis of glycosyl donors (e.g., UDP-glucose) (Table S4), thereby promoting the accumulation of glycosidic metabolites. This regulatory mechanism underlies the notably higher diversity and abundance of glycosides observed in the bulbs of the ‘Xuefeng’ cultivar. Ultimately, this cascade of changes results in the accumulation of a greater variety and quantity of glycosides in X bulbs. Notably, transcriptomic data revealed differential expression of seven glycosyltransferase genes, further supporting enhanced glycosylation capacity in X bulbs (Table S4). However, the precise molecular mechanisms underlying this pathway require further investigation. For example, it remains unclear which specific glycosyltransferases are activated, how their substrate preferences change, and how energy status precisely regulates sugar nucleotide synthesis gene expression.

### 4.3. Potential Regulatory Role of Soil Microorganisms on Glycoside Accumulation in Lv Bulbs

This study employed metagenomic analysis to distinguish rhizosphere and non-rhizosphere soil microbial community differences between the two *Lv*s and assess their potential impact on glycoside accumulation (Figure 3, Figure 4 and Figure 5). LEfSe analysis and functional prediction of differentially enriched microbial taxa indicated higher carbohydrate metabolism activity but lower glycan biosynthesis and metabolism activity in both the rhizosphere and non-rhizosphere soils of X compared to that of *Lv* (Figure 4a). We speculate that this pattern arises because glycoside accumulation is likely primarily governed by host genetic factors, while soil microorganisms may function more to supply precursor substrates. Thus, the enhanced glycoside synthesis in X is mainly driven by its genetic background, with rhizosphere and non-rhizosphere soil microbial communities synergistically promoting accumulation by modulating carbon source supply and precursor production. However, this mechanism remains hypothetical and requires further validation through functional studies, such as isolating key microorganisms and performing recolonization experiments under sterile conditions. Furthermore, VPA showed that the interaction between genetic factors and the microbial community collectively explained 86.8% of the metabolite variation, while individual factors each accounted for less than 10%. This further supports the notion that glycoside accumulation in X is co-influenced by rhizosphere microorganisms and genetic factors. Based on our results, we hypothesize that Planctomycetes bacteria in the rhizosphere and Acidobacteriia and Rhodanobacteraceae in the non-rhizosphere soil may enhance carbon metabolism, providing precursors for glycoside polymerization to the host plant, while significantly upregulated genes in the plant accelerate the conversion of glycosyl donors into active compounds like flavonoid glycosides and glucosinolates. Overall, the pattern for the bulbs’ glycoside synthesis involves microbes primarily supplying precursors, while genetic mechanisms dominate the synthesis process, ultimately leading to the significant increase in both variety and content of glycosides in X bulbs. Due to the limited number of cultivars used in this study, this hypothesis remains preliminary regarding glycoside synthesis. Future studies utilizing a broader range of *Lv* materials are warranted to further validate this mechanism.

## 5. Conclusions

The difference between L and X can be attributed to the synergistic effect between the genetic background and rhizosphere microorganisms. Transcriptome analysis identified 642 DEGs related to sugar metabolism, which were significantly enriched in pathways such as starch and sucrose metabolism, thus promoting glycosyl donor production. Microbiome analysis revealed that rhizospheric Planctomycetes and non-rhizospheric Acidobacteriia and Rhodanobacteraceae enhance precursor supply by boosting carbon metabolism. VPA confirmed that the interaction between genes and microorganisms collectively explains 86.8% of the metabolite variation. The high glycoside accumulation trait of the ‘Xuefeng’ ecotype is associated with its unique genetic mechanisms and rhizosphere microecological interactions. These findings established a theoretical framework for dissecting glycoside-polymer biosynthesis in *Lilium brownii* var. *viridulum* Baker and for the targeted breeding development of elite medicinal/edible cultivars. The models presented here provide a baseline whose broader applicability across the germplasm spectrum remains to be validated.

## Data Availability

Soil metagenomic raw sequence data support the findings of this study have been deposited into China National GeneBank DataBase (CNGBdb) with accession number CNP0007916. All other data are available from the corresponding author upon reasonable request.

## Supplementary Materials

The following supporting information can be downloaded at: https://www.mdpi.com/article/10.3390/metabo15110712/s1, Figure S1: Volcano plot of differential metabolites in the bulbs of two *Lv*s; Figure S2: Differential fold changes in monosaccharides in the bulbs of two *Lv*s; Figure S3: KEGG enrichment analysis of differential metabolites in two *Lv*s; Figure S4: Differences in glycoside content and secondary metabolites in the two *Lv*s; Figure S5: Transcriptomic PCA of two *Lv*s; Figure S6: KEGG enrichment analysis of differential genes between two *Lv*s; Figure S7: GO enrichment analysis of differential genes between two *Lv*s; Figure S8: Bacterial composition at the phyla level; Figure S9: Bacterial composition at the genus level; Figure S10: KEGG enrichment analysis of differential microorganisms; Table S1: Identification of differential metabolites between X and L. xf, X; ly, L; Table S2: Quality control information for the transcriptome data of X and L. xf, X; ly, L; Table S3: 642 genes were directly enriched in the KEGG pathways for carbohydrate metabolism in X and L; Table S4: Analysis of the top 10 upregulated and top 10 downregulated differentially expressed genes associated with glycoside metabolism. xf, X; ly, L.
