# Supplementary material for: Multi-Omics Decoding of Potential Microbial–Genetic Synergy Underlying Polysaccharide and Glycosidic Polymer Biosynthesis in Two Cultivars of Lilium brownii var. viridulum Baker
Source: Metabolites. 2025 Oct 30;15(11):712. doi: 10.3390/metabo15110712 (PMC12654296; doi:10.3390/metabo15110712)
Supplement: Supplementary file 1 [file metabolites-15-00712-s001.zip › Additional file S1.pdf]

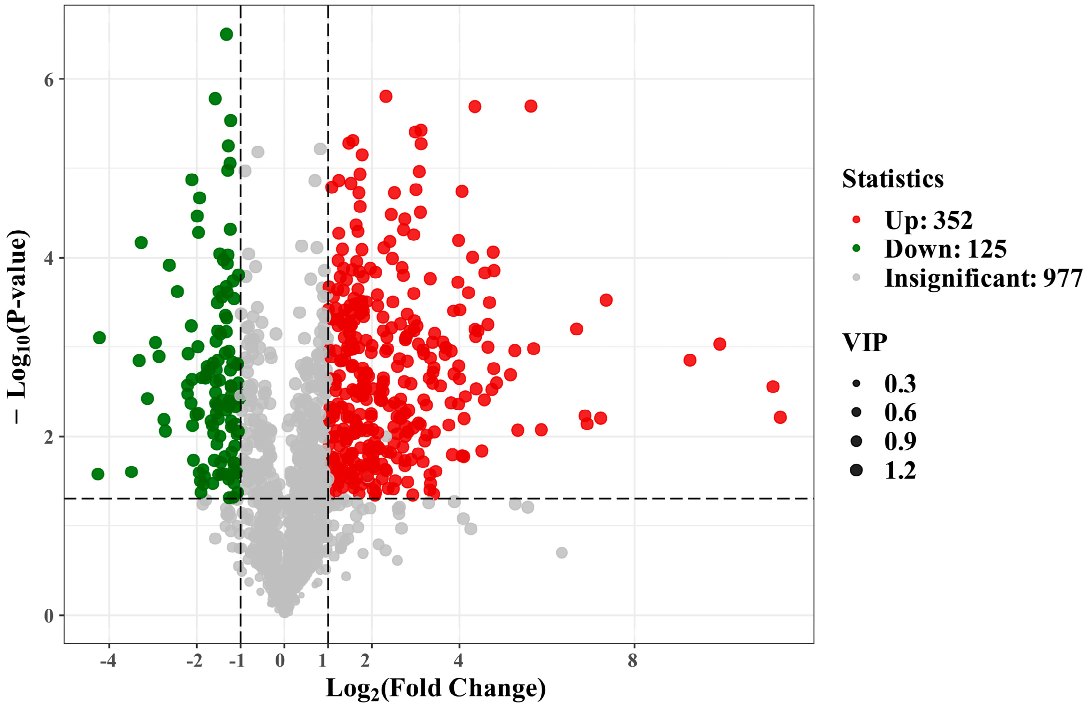

Figure S1. Volcano plot of differential metabolites in the bulbs of two *Lv*s

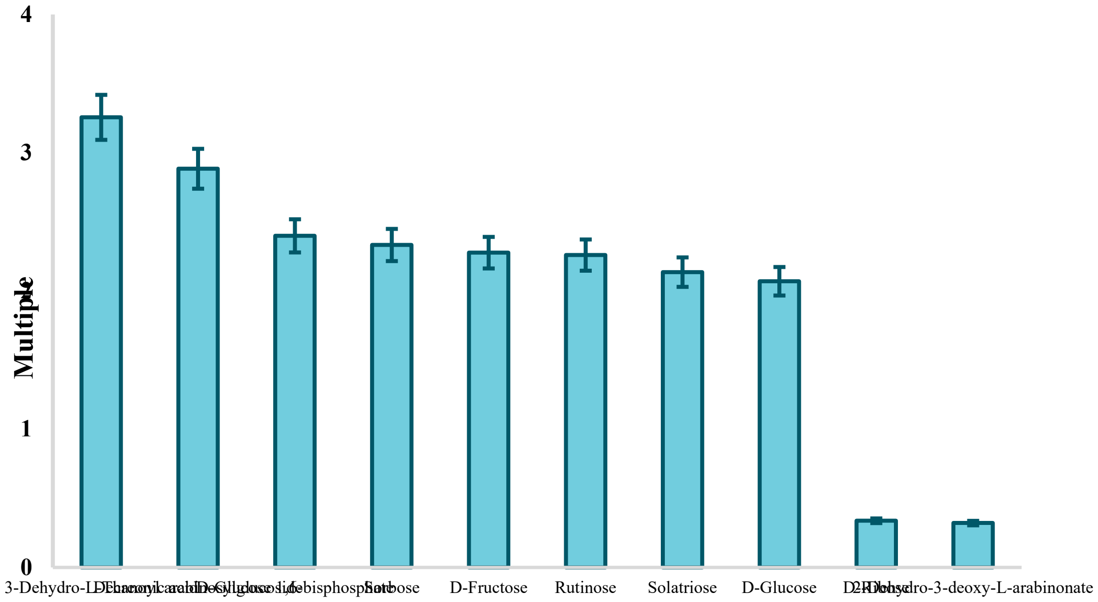

Figure S2. Differential folds of monosaccharides in the bulbs of two *Lv*s

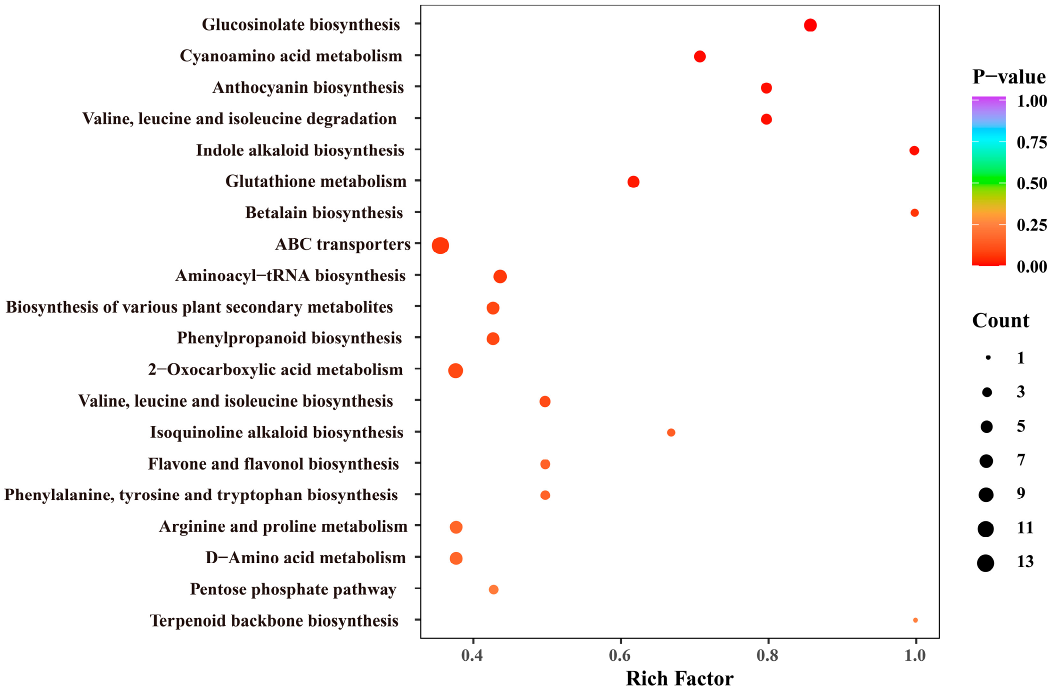

Figure S3. KEGG enrichment analysis of differential metabolites in two *Lv*s

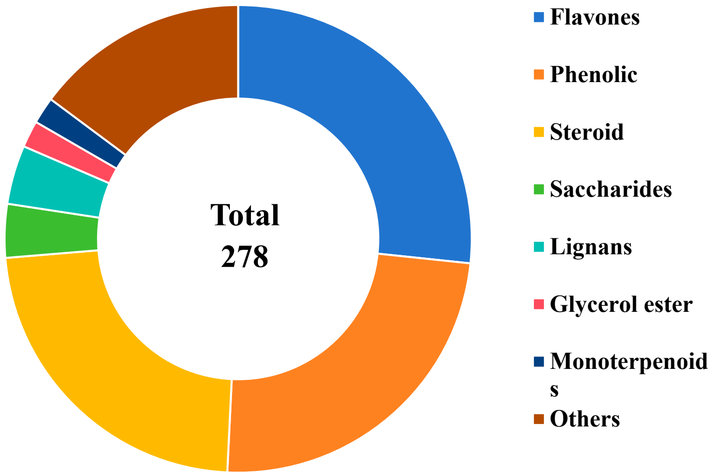

Figure S4. Differences in two *Lv*s glycoside with secondary metabolites

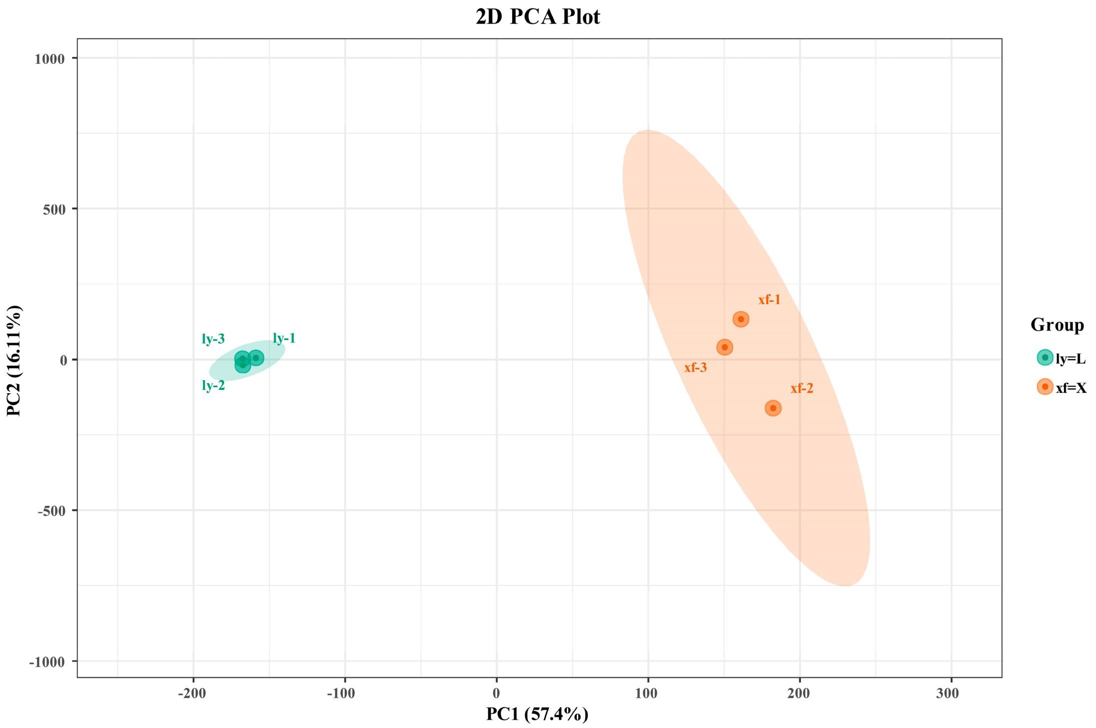

Figure S5. Transcriptomic PCA analysis of two *Lv*s. Ly (L): ‘Longya’, xf (X): ‘Xuefeng’.

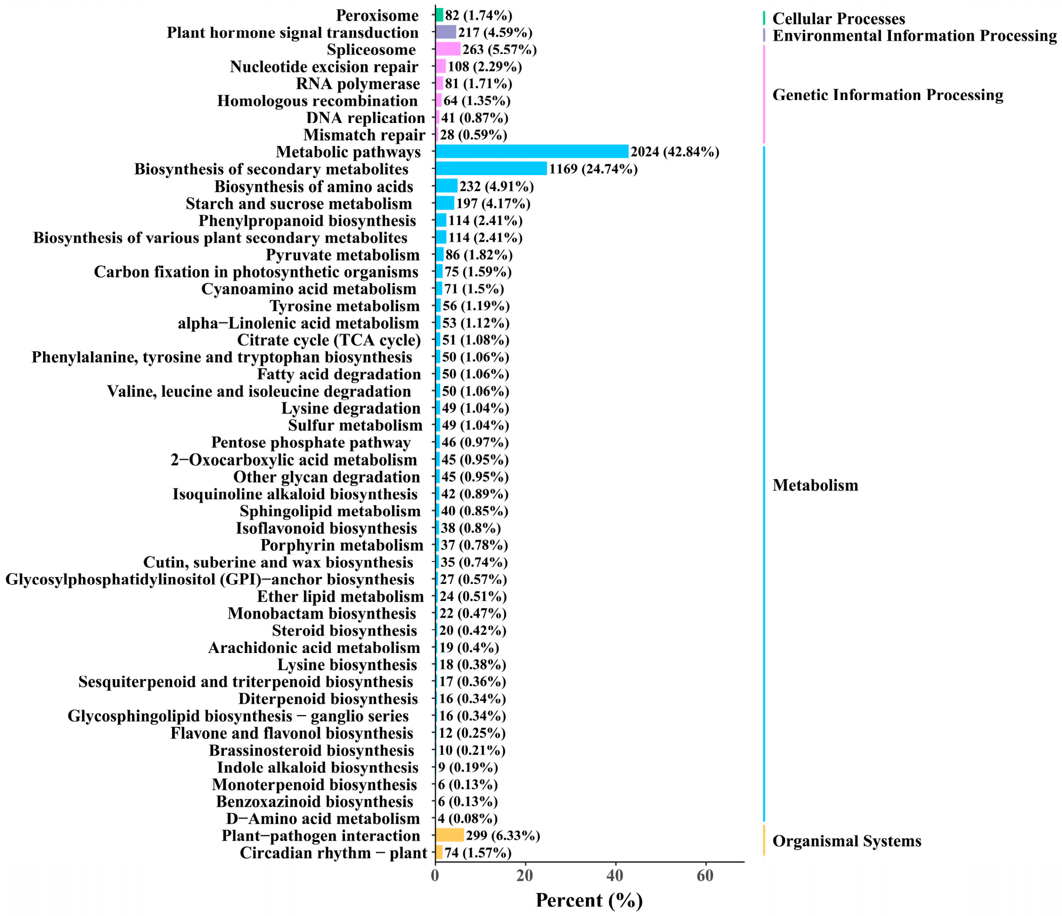

Figure S6. KEGG enrichment analysis of differential genes between two *Lv*s

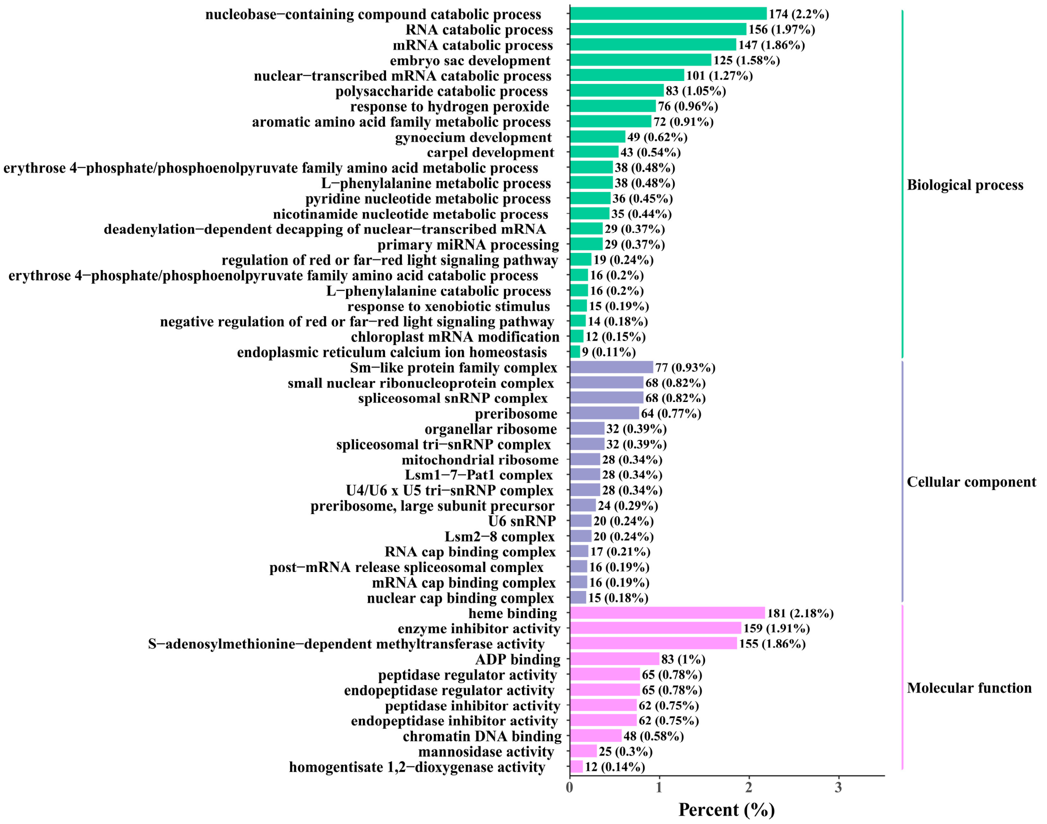

Figure S7. GO enrichment analysis of differential genes between two *Lv*s

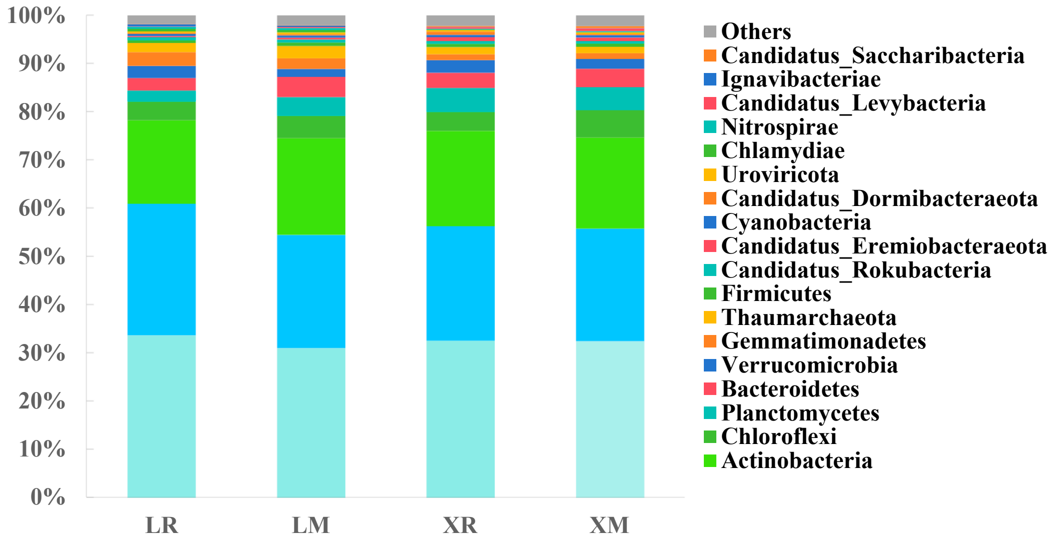

Figure S8. Bacterial composition at the phyla level. L: 'Longya' , X: 'Xuefeng'. R: Rhizosphere soil, M: Non-rhizosphere soil.

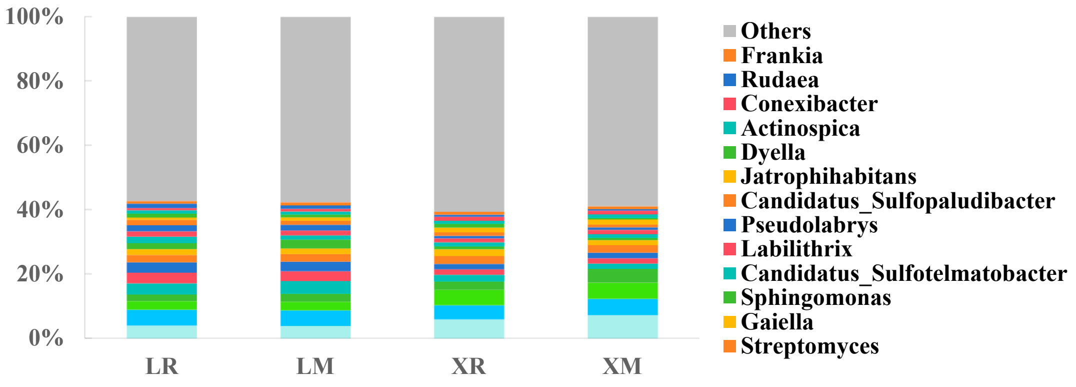

Figure S9. Bacterial composition at the genus level. L: 'Longya' , X: 'Xuefeng'. R: Rhizosphere soil, M: Non-rhizosphere soil.

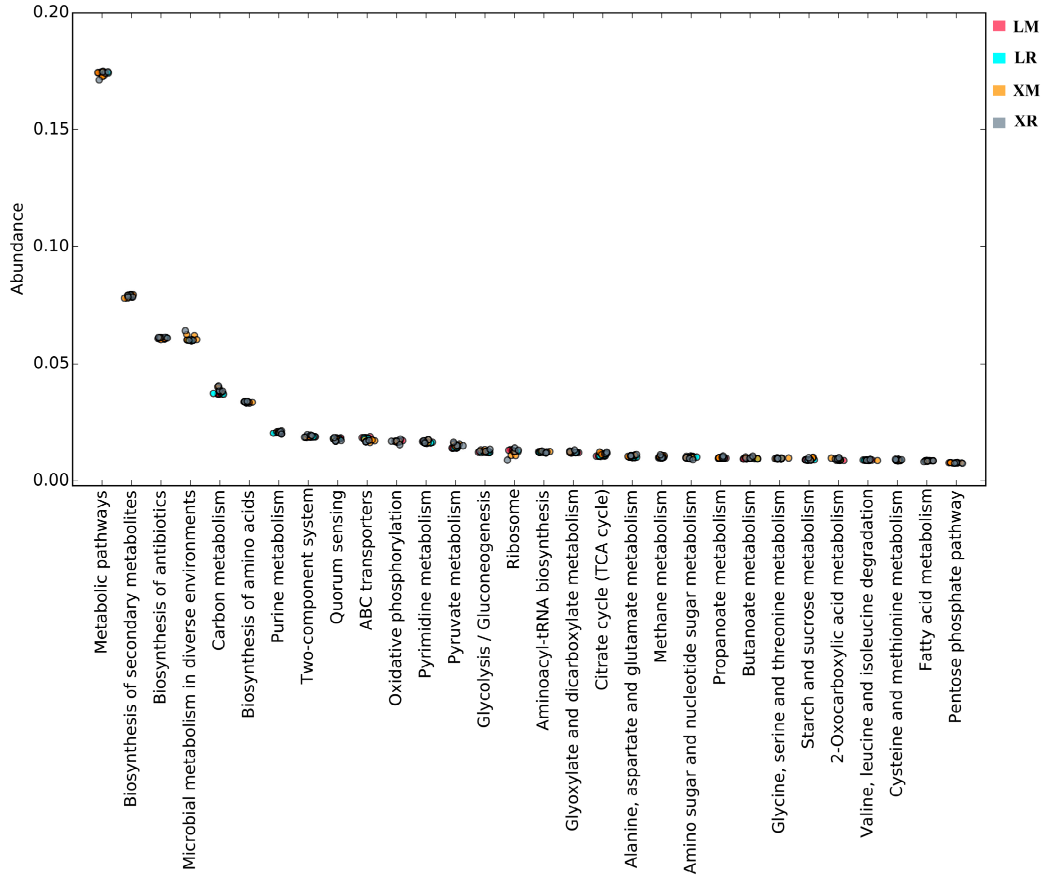

Figure S10. KEGG enrichment analysis of differential microorganisms. L: 'Longya' , X: 'Xuefeng'. R: Rhizosphere soil, M: Non-rhizosphere soil.
